# Case of Supposed Uterine Cancer, in Which a Sponge Was Retained in the Vagina for Two Years

**Published:** 1876-09

**Authors:** Edward W. Jenks

**Affiliations:** Professor of Diseases of Women, Etc., Detroit Medical College


### A CASE OF SUPPOSED UTERINE CANCER, IN WHICH A SPONGE WAS RETAINED IN THE VAGINA FOR TWO YEARS.

By EDWARD W. JENKS, M.D.,

Professor of Diseases of Women, Etc., Detroit Medical College.

I was summoned, not long since, to see a patient who was supposed by her friends and medical attendant to have cancer of the uterus, from a sanious vaginal discharge which had been profuse and offensive for over a year. It is but just to my friend, who called me in consultation, to say that he had formed no opinion of the case from physical examination, as he postponed that until I saw the patient with him.

The patient, Mrs. ——, sixty-two years of age, stated that she had ceased menstruating twelve years previous; that she was the mother of several children, and had always possessed good health until the present difficulty. The discharge she said was not only very offensive, but exhausting and very irritating. Upon examination the external genitalia were seen to be deeply excoriated and the inner part of the thighs in a similar condition. Carrying a finger into the vagina, I could distinctly feel at the uterine extremity a soft immovable mass, unlike any morbid growth I had ever before encountered. I then inserted a speculum and saw that it was a sponge quite firmly held in position. A portion seemed almost encysted, so that the force required for its removal by dressing forceps tore it into pieces. As the sponge was removed the atrophied, uterine neck, and the vagina surrounding it, were seen to be ulcerated and bleeding surfaces.

The patient informed me that she had formerly been troubled with "falling of the womb," for which she had been in the habit of having sponges inserted; that the last one was put in by a physician two years before, which she was quite sure she had afterwards removed. Soon after this she began to have the offensive discharge which occasioned my visit. It is probable that she may have removed a portion of the sponge that was inserted, or may have removed none, but thought she had. Only for the peculiar condition of the neck of the uterus and the vagina, occasioned by the senile changes of these organs, it could be hardly possible for the sponge to be so firmly retained such a length of time in the superior portion of the vagina; and at no other time of life than after the menopause, could a foreign substance like a sponge be in constant apposition with the neck of the uterus for two years and produce such slight derangement. It is needless to add that with the removal of the sponge the patient made a rapid recovery with no other treatment than the use of detergent vaginal washes.

